# Violence, land conflicts and the dynamics of the agrarian economy in the Brazilian Amazon

**DOI:** 10.1590/0102-311XEN064024

**Published:** 2025-03-24

**Authors:** Ana Paula Dal’Asta, Wellington Augusto Araújo Farias, Antonio Miguel Vieira Monteiro, Silvana Amaral, Alexandre Bahia Gontijo, Danilo Araújo Fernandes, Maria Isabel Sobral Escada

**Affiliations:** 1 Instituto Nacional de Pesquisas Espaciais, São José dos Campos, Brasil.; 2 Faculdade de Tecnologia, Jacareí, Brasil.; 3 Laboratório de Produtos Florestais, Brasília, Brasil.; 4 Universidade Federal do Pará, Belém, Brasil.

**Keywords:** Violence, Rural Economy, Amazonian Ecosystem, Violencia, Economía Rural, Ecosistema Amazónico

## Abstract

In the Amazon, the land market is imposed by the conversion of the forest into land for economic purposes. Part of this land is the result of illicit actions, using different levels of violence and producing changes in the forest landscape. We argue that conflicts over land are signs in the territories of the action of mechanisms to generate land as a commodity in the region. Based on the situations of land conflicts mapped by the Pastoral Land Commission in the period from 2012 to 2021 and the analytical categories of the agrarian economy (technological trajectories, TTs) for 2006 and 2017, an analysis was built at the municipal level based on two periods. From 2012 to 2017, we sought to identify and characterize the territories where these mechanisms were present, observed in conjunction with the state transitions of the TTs from 2006 to 2017. From 2018 to 2021, we sought to identify processes and trends for the most recent period. Municipalities that converged to economies based on livestock and grain farming systems concentrated more than 60% of the conflicts that occurred in the decade. The results show the persistence of conflicts in historical areas, such as in the southeast of Pará, and in new borders, such as in municipalities on the Amazonas, Rondônia and Acre borders. There is also the internalization of violence towards the western Amazon, in municipalities with economies based on the biome in different arrangements, revealing new fronts of interest for the land resource. The results contribute to the debate on violence in the Amazon, based on the choices for the agrarian development model, which can result in illnesses of individuals and communities trapped in disputes, to control the ways of living and producing.

## Abstract (es)

En la Amazonia, el mercado de tierras se impone convirtiendo la selva en tierras para el mercado. Parte de estas tierras es resultado de actividades ilícitas, con el uso de diferentes niveles de violencia, y produce cambios en el paisaje forestal. Sostenemos que los conflictos por la tierra son señales en los territorios de acción de mecanismos generadores de tierra, como mercancía, que operan en la región. Con base en las situaciones de conflicto por tierra mapeadas por la Comisión Pastoral de la Tierra en el período del 2012 al 2021 y las categorías analíticas de la economía agraria (trayectorias tecnológicas, TT), para el 2006 y el 2017, construimos un análisis a nivel municipal con base en dos períodos. Del 2012 al 2017, buscamos identificar y caracterizar los territorios en los que estos mecanismos estaban presentes, observándolos junto con las transiciones estatales de las TT del 2006 al 2017. Del 2018 al 2021, buscamos identificar procesos y tendencias para el período más reciente. Los municipios que convergieron hacia economías basadas en sistemas patronales de ganadería y agricultura de granos concentraron más del 60% de los conflictos que tuvieron lugar en la década. Los resultados muestran la persistencia de conflictos en áreas históricas, como en el sudeste del Pará, y en nuevas fronteras, como en municipios en la frontera de Amazonas, Rondônia y Acre. También está la internalización de las violencias hacia la Amazonia occidental, en municipios con economías basadas en el bioma en diferentes arreglos, revelando nuevos frentes de interés para el recurso tierra. Los resultados contribuyen al debate sobre las violencias en la Amazonia, con base en opciones de modelo de desarrollo agrario, que pueden resultar en la enfermedad de individuos y comunidades atrapados en disputas para el control de las formas de vida y de producción.

## Introduction

Minayo’s text ^1^ presenting the concepts and typologies of violence opens the collection Impacts of Violence on Health ^2^ and helps to situate the path that led the World Health Organization (WHO) in 1996 and, in 2001, the Brazilian Unified National Health System (SUS, acronym in Portuguese), to include violence in the Health agenda ^3^. For the WHO, violence comprises “*the intentional use of physical force or power, real or threatened, against oneself, against another or against a group or a community, which results or has a high possibility of resulting in injury, death, psychological damage, developmental disability or deprivation*” ^4^ (p. 5). 

In 1971, Dom Pedro Casaldáliga denounced, for the first time in a pastoral letter ^5^, the various forms of violence against communities that were in defense of the right to preserve the way of living and producing present in the Amazon for centuries ^6^ ^,^ ^7^ ^,^ ^8^: the access to land. This situation of violence persists in the region, which, in 2022, recorded approximately 60% of the conflicts in the countryside that occurred in Brazil ^9^. In 2017, four of the five massacres in the countryside occurred in the Amazon and resulted in the deaths of 25 landsmen ^10^. The records of the Pastoral Land Commission (CPT, acronym in Portuguese) point to the intensification of these conflict situations in recent years and the need to think about the dimension of the suffering of real people behind the numbers ^9^. In addition to damages and deaths, which are more visible impacts, conflicts also manifest themselves through threats against people or occupations, which also result in indirect impacts, affecting the physical and mental well-being of individuals and populations and increasing the challenge to public health in the region ^11^ ^,^ ^12^.

The literature on agrarian conflicts in the Amazon is broad and, in general, describes conflicts as a complex and multifaceted issue. Conflicts are portrayed as a consequence of the combination of factors related to the land structure, the narrative about “modernization” of the countryside, the developmental strategies of the three spheres of government and the set of illicit activities associated with economic activities that, in their chains, involve land grabbing, illegal mineral and logging ^13^ ^,^ ^14^ ^,^ ^15^ ^,^ ^16^.

The economist Francisco de Assis Costa, anchored in rural sociology, in the economic history of the Amazon and from empirical and case studies in the Amazon itself, centralizes this debate from the perspective of the regional agrarian economy, by establishing land conflicts as the most visible dimension of the antagonism between the two main projects in dispute for rural-based development in progress in the Amazon ^6^ ^,^ ^17^. These projects are essentially based on the evolution of two paradigms associated with the technical production systems present in the Amazonian agrarian economy: one whose main techno-productive foundation is the conservation and maintenance of the integrity and diversity of the biome, and the other that is supported by the homogenizing pattern, which has the exploitation of the biome’s natural resources as its main developmental strategy.

In essence, these two patterns mobilize issues related to land use, workforce organization, and the technological solution package and use of internalized biodiversity in their production systems. When combined with different agents with specific economic rationalities (peasant and capitalist), several rural production systems emerge that can be organized to establish, in the context of the analytical economic category developed by Costa ^6^ ^,^ ^18^, the technological trajectories or rural technical-productive trajectories (TTs, acronym in Portuguese), a set of six trajectories present in the Amazonian agrarian economy.

Although with internal nuances to the patterns of peasant and capitalist rationality, the basis of the homogenizing technological package that drives (based on mechanical-chemical-genetic solutions) the rural productive dynamics in the Amazon ^19^ represents the main driver of forest cover changes and the main stimulus to agrarian conflicts. From this perspective, the forest resource and the population that resides in the region are seen as an obstacle to the expansion of agricultural production in the region. The idea is a way of producing that uses natural resources as a still-life ^20^ ^,^ ^21^.

In the alternative paradigm, in turn, there is a greater variety of technological solutions, allowing greater efficiency in the use of the forest resource and the cultural values already established by the traditional communities in the region, which is incorporated into the modes of production of the new economic agents that transfer to the biome, configuring a model of techno-productive evolution similar to agroforestry systems. The biome is seen in an integrated way, as a productive input, in which the way of producing depends on living nature, that is, on the biome in its greatest integrity. Structural and cultural diversity is a fundamental part of the productive and economic organization of these systems considered socially and ecologically healthier and more resilient ^22^ ^,^ ^23^, whose production zones cannot be considered pre-capitalist ^6^.

In agrarian economy, these two projects or paradigms represent different models of production, reproduction, and distribution of available assets, and the TTs associated with them are co-occurring, sometimes in competition and sometimes in collaboration. For the homogenizing production model, the transformation of land into a commodity represents an essential step in the entire production process, as it is seen as a productive input. Thus, a land market is imposed, treating it as a commodity of a generic nature, whose production occurs through the conversion of the original forest, a public good, into private assets ^8^ ^,^ ^24^. It consists in the forest, firstly, being transformed into forest land, which is the primary input for the production of forestless land: “land for agriculture” or “land for livestock” − constituting land for the market ^17^. On the other hand, in the dynamics associated with peasant systems, the forest land is a means of production guaranteeing the economy of the peasant production unit, so that there is a non-capitalist economy working within a capitalist economy ^25^ ^,^ ^26^.

Thus, the demand for forestless land (land commodity) becomes a constitutive and mobilizing element of the dynamics of the Amazonian agrarian economy, establishing a point of origin for agrarian conflicts. Such economy fosters land concentration and subjects the peasant population to various forms of violence and land insecurity ^27^. Reiterating, Costa et al. ^28^ analyzed data from the federal police on the Amazon from 2016 to 2020, on the prevention of environmental crimes, and observed that 47% of the investigated properties resulted from land grabbing and 60% had deforestation or illegal logging, demonstrating that this land market is interconnected to the set of illegal activities, and, therefore, to violence and human rights violations. This study assumes that part of the land accommodated in the agrarian system comes, at some level, from illicit activities, i.e., from spoliation forms of land appropriation, with the use of violence, resulting in transformations of the forest landscape.

One way to recognize the main land-generating mechanisms in progress in the territory where they operate is based on the conflict situations mapped by the CPT, which since 1985 has recorded the annual accumulation of situations of violence involving the various categories of peasant in Brazil ^29^. Based on this collection, it is possible to obtain the conflicts anchored in the territory, arising directly from the production chain of these lands.

This study seeks to answer, using an approach to the construction of empirical evidence through the integration of spatial databases, how situations of conflict over land signal the action of land-generating mechanisms operating in the Legal Amazon. Our cutout for this study is the recording of land conflicts that occurred in the last decade (2012 to 2021) in the region. The analysis covers two periods. In the first, from 2012 to 2017, the territories where the land-generating mechanisms were active are identified and characterized. The state associated with the dynamics of the TTs system in 2017, through a matrix of transitions from 2006 to 2017, is observed in conjunction with the conflict data. In the second period, 2018 and 2021, we show processes and trends in land conflicts for the most recent period, considering only the state of the TTs system in 2017.

Thus, we analyzed two territorial dynamics in the regional space: the dynamics of the occurrence of land conflicts and the dynamics of the TTs system, forming an empirically based analytical instrument. The results contribute to the debate on violence in the Amazon, based on the choices made by the agrarian development model. Together with the environmental outcome, observed by deforestation, land conflicts are a health outcome, revealing illnesses of individuals and communities trapped in a series of disputes, asymmetric in power, for the control of the ways of living and producing in a biome that historically evolves with the presence of ancestral communities and populations.

## Material and methods

### Study area

The Legal Amazon represents 58.93% of the Brazilian territory and includes 772 municipalities in the North, Northeast, and Central-West regions. With 5 million km^2^, the Legal Amazon is home to 26,644,564 inhabitants (13.12% of the Brazilian population) ^30^ and great socioenvironmental diversity ^7^ ^,^ ^8^. From 2012 to 2021, the nine Legal Amazon states concentrated 63% of the 10,318 land conflicts that occurred in Brazil, which involved approximately 700,000 families and resulted in 258 deaths ^29^. In this period, conflicts in the region intensified, culminating in 1,042 land conflicts in 2020, the highest number recorded in CPT’s annual historical series. At the same time, the region observed an increase in the deforestation rate, reaching almost 13,000km^2^ in 2021and 2022 ^31^, and the expansion of agribusiness, with a 286% growth in soybean planted area between 2006 and 2017 ^32^.

## Materials

The main database of this work is the conflict situations documented by the CPT and available in the Dom Tomás Balduíno Documentation Center Collection (CEDOC, acronym in Portuguese) ^29^.For the CPT, conflicts are actions of resistance and confrontation that happen in different social contexts in the rural area involving the struggle for land, water, rights, and the means of work and production, and occur between social classes, among workers, or due to the absence or mismanagement of public policies ^29^. Because this basis is continuously updated, the total number of conflicts may differ from that disclosed in the annual reports.

On the CPT basis, records are categorized into land, water, and labor conflicts. Only land conflicts were analyzed, i.e., actions of resistance and confrontation for the possession, use and ownership of land and access to natural resources, involving the most diverse categories of peasant ^9^. Conflicts are indexed by name and each action of confrontation or resistance is considered an occurrence. Thus, in one area, more than one conflict situation may occur throughout the year. Each conflict is also described in terms of the number of families affected, the type of violence suffered, and the social agents involved. The municipality of origin of the occurrence of each conflict was considered for spatial indexing and conflicts that occurred between 2012 and 2021 were selected.

The second database used refers to the territorialized base of the Amazonian agrarian economy mediated by the TTs. To identify TTs, Costa ^18^ developed a method based on multivariate regressions and principal component and factor analysis techniques applied to data extracted from the last three Brazilian Agricultural Census (1995, 2006 and 2017). From this approach, six types of TTs emerged for Legal Amazon municipalities, categorized as peasant (TT1, TT2 and TT3) and capitalista (TT4, TT5&6 and TT7) and characterized in terms of modes of production, types of activities, workforce and package of technological solutions (Box 1). The percentage shares of TTs in relation to the agrarian component of the value of municipal production are available in Costa ^33^. In this study, we adopted the dominant TT in each municipality, that is, the TT that contributed more than 50% of the total value of the contribution derived from the rural economy to the Gross Production Value in 2006 and 2017.

**Box 1:** Description of the set of technological trajectories (TT) present in the Amazonian agrarian economy.

AGENTS	DESCRIPTION	TT	PRODUCTIVE SYSTEMS
Peasant	Peasant-based production systems run by “family-based” arrangements	TT1	Systems that converge to small-scale permanent crops (cocoa, pepper, coffee) and temporary crops (cassava, corn, rice and beans)
		TT2	Agroforestry systems
		TT3	Small-scale beef and dairy farming and temporary and permanent crops
Capitalist	Establishments managed by the “agribusiness” or “salaried” regime, where microeconomic decisions are aligned with large markets and commodity trade	TT4	Large-scale livestock farming and production of crops for animal consumption
		TT5&6	Large crops of permanent crops, planted forests and technified forestry
		TT7	Intensive large-scale temporary crop systems (soybeans, rice, corn, etc.)

### Methodological procedures 

In this exploratory territorial-based study, the spatial units of analysis are the municipalities of the AML, which were associated with situations of land conflicts between 2012 and 2021 and with the analytical categories of agrarian economics (TTs) for the years 2006 and 2017. 2017 was adopted as a reference year, given the most recent classification of TTs, dividing the decade into two periods of analysis. From 2012 to 2017, we sought to identify and characterize the territories where the land-generating mechanisms operated in the period that culminated in the TTs found in 2017. From 2018 to 2021, we sought to identify the processes and trends associated with land conflicts, keeping as a reference the categorization of TTs in 2017, considering that these conflicts originate in these categories or are produced by the transitions that involve them.

The evolutionary dynamics of the TTs system observed the state of the system in 2006 and 2017, seeking to identify the transitions of change and non-change for each municipality. That is, whether the dominant TT in 2006 remained dominant in 2017 or whether there was a change of state, indicating the nature of the transition. A matrix of the state transitions of the TTs between 2006 and 2017 was constructed and the quantitative of the occurrences of aggregate conflicts for the period from 2012 to 2017 were associated with the identified transitions. For the period from 2018 to 2021, the accumulated conflicts were integrated with the state of the TTs system observed in 2017.

In addition, we explored the hypothesis of spatial dependence associated with the occurrences of conflicts over land for the periods analyzed. Indices that measure global and local spatial autocorrelation were used, through the Global and Local Moran Indices and an exploratory panel based on Local Indicators of Spatial Association (LISA) ^34^. While global indicators provide a single measure of spatial association for the entire data set, local indicators produce a specific value for each municipality, allowing the identification and characterization of municipal agglomerations. LISA is a decomposition of the Global Moran index, calculated for each municipality, which allows to represent, in the form of a map, the spatial dependence structure at the local level.

This analysis results in four types of municipal spatial clusters: High-High groups municipalities with high occurrence of conflicts and whose neighborhood also has municipalities with high occurrence of these events in the same period; Low-Low groups municipalities with low occurrence of conflicts whose neighborhood also presents low occurrence in the same period, and High-Low and Low-High, indicative of municipalities with high/low occurrence of conflicts surrounded by municipalities with occurrence in the opposite situation. To obtain the indexes, the GeoDa (https://spatial.uchicago.edu/geoda) ^35^ software was used and pseudo-significance was performed with 999 random permutations.

## Results

### The geography of land conflicts in the Amazon in the 2012-2021 decade

From 2012 to 2021, CEDOC/CPT counted 6,505 land conflicts in the nine Legal Amazon states. Annually, land conflicts showed relative stability between 2012 and 2015, with an annual average of 475.5 conflicts; peak in 2016, when 764 conflicts were registered, and an annual average of 682.3 conflicts in the period between 2016 and 2018; and a high number of conflicts between 2018 and 2021, with an annual average of 852. In 2020, the number of land conflicts (1,042) was also impacted by the COVID-19 pandemic, mainly due to the omission and connivance of the State in situations of exposure and vulnerability of many families ^36^. From 2019 to 2021, 2,552 conflicts (39.2%) were counted.

Maranhão, Pará and Rondônia concentrated 57.1% (3,714 conflicts) of the total conflicts. In relative terms, over the analyzed period, the contribution of Maranhão (from 33.3%, in 2012, to 13.9%, in 2021) and Amapá (11.7%, in 2012, to 7%, in 2021), in the total conflicts registered in the Leagal Amazon, decreased, while the participation of Mato Grosso (from 5.5%, in 2012, to 12.7%, in 2021), Pará (from 17.5%, in 2012, to 22.6%) and Roraima (no conflict in 2012 to 7.4% in 2021), increased. In Roraima, the peak of conflicts in 2021, which represented 10.9% (113) of the total occurred in the Legal Amazon, refers to violence against indigenous territories and peoples.

Regarding the person responsible for the conflicts, according to the CPT categorization ^29^, farmers accounted for 31.9% (2,073), followed by land grabbers with 17.5% (1,140), and entrepreneurs with 16.1% (1,047) of the conflicts. Farmers are the category that caused the most conflicts in most states: Acre (64.1%, 378), Rondônia (51%, 419), Mato Grosso (44.4%, 283), Tocantins (31.3%, 136), Maranhão (28.3%, 469), and Amazonas (26.3%, 103). It also had a significant participation in Pará, accounting for 20.2% (249) of the occurrences. Land grabbers caused about 30% of conflicts in Tocantins (132) and Pará (366), 21.7% (85) in Amazonas, and about 15% in Mato Grosso (107), Rondônia (101) and Maranhão (249). Conflicts caused by entrepreneurs were especially relevant in Amapá, representing 67.9% (381) of conflicts, and in Maranhão (23.6%, 392). Conflicts caused by the state and agents of the law, in their different spheres, occurred in all states, being more significant in Roraima (26.1%) and Tocantins (25.1%). For Roraima, the data reflect the conflicting relationship between mining and indigenous peoples and their territories: 58% of the conflicts that occurred in the state were caused by miners and in 72.7% the indigenous people were the victims. Across the Legal Amazon, indigenous people were targeted in 1,216 conflicts (18.7%), especially in Pará (299), Mato Grosso (290) and Maranhão (146).

Squatters represent the category that suffered the most violence in the analyzed period: 1,679 conflicts (25.8%), of which: 60.6% (340) of the total conflicts occurred in Amapá, 40% (236) in Acre and 37.6% (623) in Maranhão. Conflicts involving the landless totaled 1,249 occurrences (19.2%), of which 40.3% (504) were in Pará and 29.2% (365) in Rondônia. In Maranhão, 37.6% of the conflicts recorded were against quilombolas, and in Acre, rubber tappers were victims in 48% of the occurrences.

### The agrarian economy and land conflicts in the Amazon

**Figure 1:**
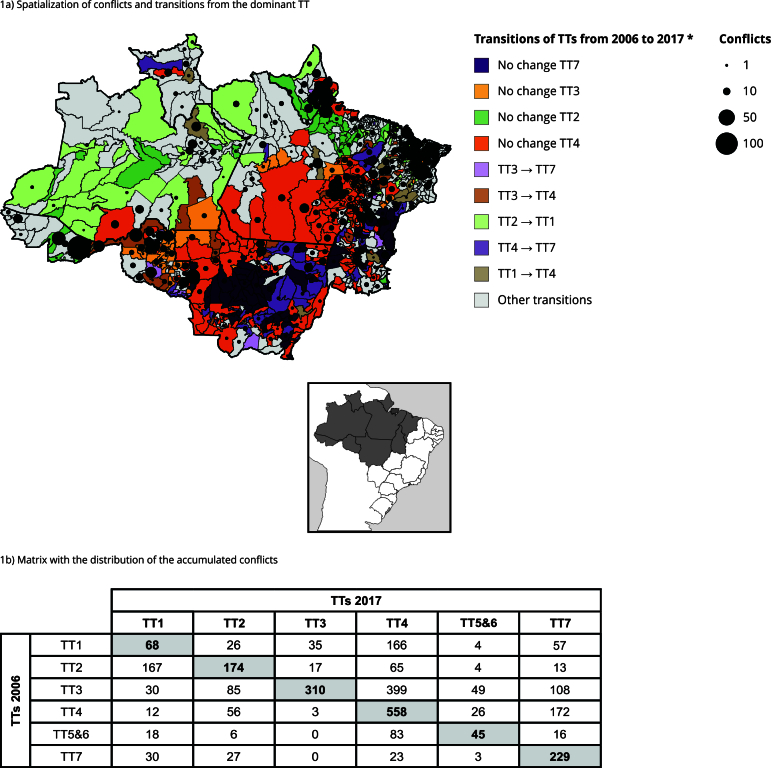
Spatialization of conflicts (2012 to 2017) and transitions from the dominant technological trajectories (TT) (2006 to 2017), and matrix with the distribution of the accumulated conflicts (2012 to 2017) by the types of state transitions of the dominant TT (2006 to 2017).

Between 2012 and 2017, 3,326 (51.12% of the total in the decade) occurrences of land conflicts were recorded in the Legal Amazon distributed in 386 municipalities. Of these, 1,700 conflicts occurred in municipalities with changes in the state of the dominant TT from 2006 to 2017, which represent 56.04% (436) of the total municipalities included in the database produced by Costa ^33^ (Figure 1a).

* Transitions with the highest number of occurrences of land conflicts (2012 to 2017).

TT7, associated with large-scale agriculture, expanded its domains by more than 70%, from 102 (13%) in 2006 to 176 (23%) municipalities in 2017, while the expansion of TT4, large-scale livestock, was almost 20%, from 216 (28%) to 259 (33%) municipalities. Such TTs were maintained in more than 65% of the municipalities. The transitions associated with these two TTs, whether in a situation of permanence (no change) or change, concentrated 61.25% (1,889) of the land conflicts recorded between 2012 and 2017 (Figure 1b). Of this amount, 558 (29.5%) are associated with municipalities that do not change from TT4, 399 (21.12%) in transitions from TT3, a peasant with specificity in livestock production, to TT4, a livestock capitalist that operates in direct competition with TT3, and 229 (12.12%) in municipalities that do not change from TT7. These transitions occur mainly along a continuous strip, covering the south of Maranhão, south-southeast of Pará, Tocantins, Mato Grosso, Rondônia to the east of Acre (Figure 1a).

On the other hand, the transitions related to peasant TTs accounted for 34.5% (1,064) of the conflicts of the period and three transitions stand out: no change from TT3 with 310 registered conflicts (29.1%; 41 municipalities), no change from TT2, peasant of agroforestry systems, with 174 conflicts (16.4%; 43 municipalities), and change from TT2 to TT1, peasant with specialized agriculture, with 167 conflicts (15.6%; 68 municipalities).

**Figure 2:**
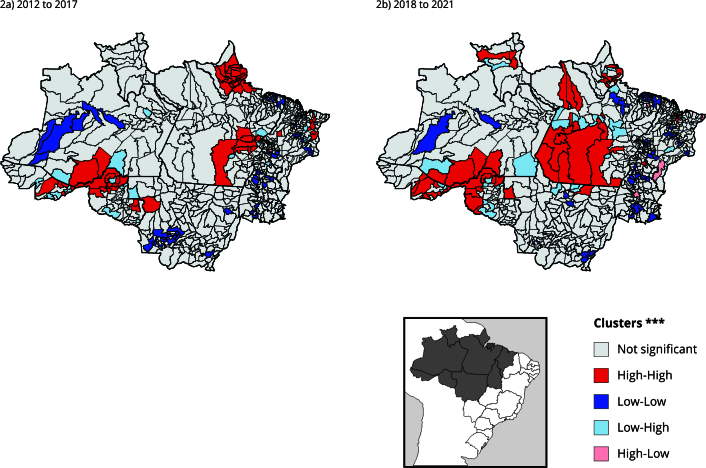
Spatial clusters of municipalities regarding the occurrence of conflicts for 2012 to 2017 * and 2018 to 2021 **.

Some of these transitions emerged in the spatial groupings of High-High municipalities for the occurrence of conflicts, in the period from 2012 to 2017, such as in the AMACRO region (acronym in Portuguese for the border region of Amazonas, Acre and Rondônia) and in southeastern Pará (Figure 2). AMACRO, most recently named the Abunã-Madeira Sustainable Development Zone (ZDS) ^37^, is one of the main active and recent frontiers of deforestation in Legal Amazon ^38^, with processes of deterritorialization of traditional peoples ^39^. The southeastern Pará, especially São Félix do Xingu, represents the persistence of violence in historical borders ^14^. Conflicts associated with the constitution of private property through the land grabbing and expansion of agribusiness and the implementation of projects, such as hydroelectric dams along the Araguari River (Amapá State), formed the grouping of High-High municipalities in Amapá and northern Pará. Municipalities that converged to TT7 in 2017, such as Tartarugalzinho, Ferreira Gomes and Itaubal (Amapá State), as well as municipalities that remained in peasant TTs, such as Mazagão, Macapá (Amapá State) and Afuá (Pará State), are in this grouping.

* (I: 0.206; p < 0.001); ** (I: 0.148; p < 0.001); *** Significant at 95%.

In the following period (2018 to 2021), five spatial groupings of High-High municipalities emerged (Figure 2). Again, AMACRO and São Félix do Xingu stand out, whose grouping expands by incorporating municipalities such as Altamira, Novo Progresso, Itaituba and Jacareacanga (Pará State). The grouping in Amapá persists, with fewer municipalities than in the previous period. In the northwest of Pará, Óbidos, Monte Alegre and Alenquer emerge as High-High, due to violence against quilombola populations and their territories. The High-High cluster in Roraima is explained by conflicts in indigenous territories especially from 2020. In both periods, the persistence of the Low-Low cluster in Amazonas, in the municipalities of Jutaí and Codajás, stands out.

**Figure 3:**
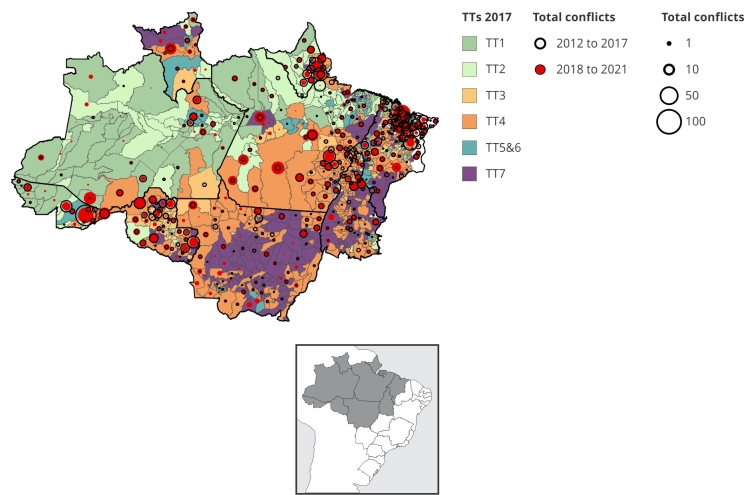
Distribution of land conflicts by municipality accumulated for the periods from 2012 to 2017 and 2018 to 2021 and the state of technological trajectories (TT) in 2017.

For the period from 2018 to 2021, 3,179 conflicts (48.87% of the total) were recorded, distributed in 396 municipalities (Figure 3). Municipalities whose dominant TT state in 2017 was peasant, accounted for 35.8% of conflict occurrences. Municipalities with dominant TT1 recorded 379 conflicts (11.9%); in TT2, 315 conflicts (9.9%), while in TT3, 445 (14%) conflicts were recorded. The municipalities associated with TT4 concentrated almost 40% (1,231) of the total conflicts recorded in the Amazon. The municipalities where TT5&6 is dominant were associated with 106 conflicts (3.3%) and those of TT7 were associated with 574 conflicts (18.1%). 

The integrated analysis of the accumulated conflicts in the municipalities in the two periods reveals three ongoing dynamics. The first refers to the dispersion of conflicts, which between 2012 and 2017 were concentrated in municipalities mainly in Amapá, Maranhão, southeastern Pará, Rondônia and Acre. The number of municipalities with land conflict records increased from 386 to 396 municipalities in the second period. Following the dispersion of conflicts, there is an internalization of violence towards the western Amazon. There are municipalities with a high number of occurrences in western Pará and Roraima states, as well as the occurrence of conflicts in new areas in municipalities in the western portion of the Legal Amazon, especially in the interior of the State of Amazonas. The third dynamic is associated with the intensification of the occurrence of conflicts in municipalities in Mato Grosso, where large-scale grain production systems predominate.

## Discussion

In the Amazon, the composition of social actors involved in situations of violence in each state varies depending on the context and historical-geographic process of occupation, whose health outcomes transcend merely clinical aspects, threatening populations and their ways of living and (re)producing. The diversity of illicit agents promoting violence includes militias, traffickers and gunmen ^29^. To Costa et al. ^28^, the land market in the Amazon is intrinsically linked to what has been called the “pillage coalition”, that is, a historical alliance between land elites, powerful economic actors, influential politicians and criminal organizations to exploit resources in the region, from legal and illegal activities, with various forms of violence. Schmink et al. ^14^ offer, from the municipality of São Félix do Xingu, an overview of conflicts and transformations at the frontier, since 1970, produced by the interaction between a set of evolving social actors with debates and policy changes at different levels.

Bruno ^40^ reflects on the guise of “modernization” and the incorporation of new symbols in the narratives of agrarian elites, while remaining inscribed in the same space of references and meanings, which include violence, environmental crime, profit and land concentration. In the period analyzed, municipalities dominated by agricultural capitalist systems were responsible for almost 4,000 land conflicts (61.5% of the total), mainly concentrated in regions dominated by large-scale livestock (TT4), where almost 50% (37,417.05km^2^) of deforestation occurred in Legal Amazon in the same period. TTs 4 and 7 have a greater weight in the land dynamics in the Legal Amazon: together they represented 78% (12.3 million hectares) of the land additions (commodity) recorded in the 2006 and 2017 agricultural censuses ^17^.

Municipalities dominated by TT4 are also associated with areas of persistence of conflicts identified by spatial clusters. These areas occur on historical frontiers, such as in the south-southeast of Pará ^14^, as well as on new fronts, such as AMACRO ^39^. Comprising 32 municipalities, AMACRO was responsible for almost 30% of the deforestation detected between 2012 and 2022 ^31^. This region is also the target of land grabbing and projects for the expansion of agribusiness, articulated by entrepreneurs in the sector in partnership with the State ^38^. As with AMACRO, the south-southeast of Pará also stands out for deforestation, with municipalities among the ten largest deforesters of Amazon ^31^. The axis associated with BR-163, which encompasses part of these municipalities and is also an area of expansion of the agricultural frontier, with a recent expansion of areas for grain agriculture ^41^.

However, it is in TT7 where the clothing of modernization is especially circumscribed to narratives. Municipalities dominated by TT7 recorded about 18% of land conflicts and correspond to 11% of deforestation ^31^. In addition, in the second period there is a dynamic of intensification of occurrences in Mato Grosso, with a strong presence of production systems associated with TT7. These results corroborate a growing but not dominant literature that discusses the tensions associated with agribusiness. In this case, the low demand for labor due to the monoculture system, mechanized with the use of chemical and genetic inputs, contributes to the rural exodus, the disorderly growth of urban peripheries, the reduction of traditional food production capacity ^27^ ^,^ ^42^ and the sickening of populations resulting from the use and spread of pesticides ^43^. In the surroundings of Santarém, for example, the expansion of mechanized agriculture occurred mainly in lands of peasant and riverside production, resulting in the homogenization of the landscape and forest loss in the production areas and their surroundings ^44^.

Our results show the dynamics of internalization of land conflicts for agroforestry municipalities in the western Amazon, observed between 2018 and 2021, revealing new fronts ^45^ of interest of the agrarian economy for the land resource. In summary, these municipalities are characterized by a greater presence of forest resources, a significant stock of land without land use ^46^ ^,^ ^47^ and maintenance of economies based on the biome in various arrangements, led mainly by TT2, with low or almost zero deforestation ^31^. Thus, unlike areas where deforestation is a fait accompli, the conflict over land on these new fronts can be seen as indicative of a stage prior to the advance of the agricultural frontier, when the land market is established, but without causing significant changes in the forest landscape. Seen in this way, the conflict over land signals in the territory that there is a dispute between development models. In other words, controlling these disputes and illicit access to land at this stage is essential, under the risk of worsening violence and the process of forest loss, which has proven to be irreversible in older occupation fronts ^45^, and all the direct and indirect losses that this process triggers in the health and well-being conditions of local populations. As the agricultural capitalist systems logic expand over the peasant territories and the forest, these systems and their populations are deterritorialized, which have in the biome in its greatest integrity the means for the social reproduction of the Amazonian way of life ^48^.

Returning to the provocation “violence is harmful to health” presented in Minayo ^1^, the impacts of land conflicts in the Amazon have their most visible dimension in injuries and deaths. However, in addition to physical violence, the conflicts systematized by CEDOC/CPT also bring together threats against people and the occupation. Taken together, these violence results in varied impacts, the existence and extent of which are not fully known, which affect the quality of life and the physical and mental well-being of individuals and populations. The recent crisis in the Yanomami (Roraima) triggered by mining, even with the recognition of their identity and territories, approved more than 30 years ago, illustrates the complexity of the impacts resulting from conflicts that affect the health conditions of the population ^49^ ^,^ ^50^, whose measures to mitigate and prevent their occurrence also represent the promotion and protection of life, including for future generations.

As limitations of this study, we mention those associated with the space-temporal units of analysis and the methodological approach. The aggregation of land conflicts at the municipal level can mask local dynamics and processes that explain the occurrence of conflicts, as well as the impacts on the health conditions of local populations. Regarding the method, exploratory analysis at the municipal level can be enhanced by the inclusion of spatial data, especially related to land structure, combined in quantitative approaches. And finally, a better characterization of the conflicts in the categories that establish the peasantry, with the treatment of the CPT database, can improve the analysis of the observed dynamics and the signaling for the outcomes related to health, the loss of forest cover and the sociobiodiversity of the biome.
