# Supplementary material for: Violence, land conflicts and the dynamics of the agrarian economy in the Brazilian Amazon
Source: Cad Saude Publica. 2025 Mar 24;41(Suppl 1):e00064024. doi: 10.1590/0102-311XEN064024 (PMC11940862; doi:10.1590/0102-311XEN064024)
Supplement: Translation

# Translation: Violência, conflitos por terra e a dinâmica da economia agrária na Amazônia brasileira

**DOI:** 10.1590/0102-311XPT064024

**Authors:** Ana Paula Dal’Asta, Wellington Augusto Araújo Farias, Antonio Miguel Vieira Monteiro, Silvana Amaral, Alexandre Bahia Gontijo, Danilo Araújo Fernandes, Maria Isabel Sobral Escada

**Affiliations:** 1 Instituto Nacional de Pesquisas Espaciais, São José dos Campos, Brasil.; 2 Faculdade de Tecnologia, Jacareí, Brasil.; 3 Laboratório de Produtos Florestais, Brasília, Brasil.; 4 Universidade Federal do Pará, Belém, Brasil.

**Keywords:** Violência, Economia Rural, Ecossistema Amazônico

## Abstract

Na Amazônia, o mercado de terras se impõe pela conversão da floresta em terras para o mercado. Parte dessas terras é fruto do ilícito, com uso de diferentes níveis de violência, e produzem alterações na paisagem florestal. Argumentamos que os conflitos por terra são sinalizadores nos territórios de ação dos mecanismos geradores de terra, como mercadoria, que operam na região. Partindo das situações de conflitos por terra mapeadas pela Comissão Pastoral da Terra no período de 2012 a 2021 e as categorias analíticas da economia agrária (trajetórias tecnológicas, TT), para 2006 e 2017, construímos uma análise em nível municipal baseada em dois períodos. De 2012 a 2017, buscamos identificar e caracterizar os territórios onde esses mecanismos estiveram presentes, observando-os em conjunto às transições de estado das TTs de 2006 para 2017. De 2018 a 2021, buscou-se identificar processos e tendências para o período mais recente. Municípios que convergiram para economias baseadas em sistemas patronais de pecuária e de agricultura de grãos concentraram mais de 60% dos conflitos ocorridos na década. Os resultados mostram a persistência dos conflitos em áreas históricas, como no sudeste paraense, e em novas fronteiras, como em municípios da fronteira Amazonas, Rondônia e Acre. Há, também, a interiorização das violências em direção à Amazônia ocidental, em municípios de economias baseadas no bioma em arranjos diversos, revelando novas frentes de interesse do recurso terra. Os resultados contribuem para o debate das violências na Amazônia, a partir das escolhas pelo modelo de desenvolvimento agrário, que pode resultar no adoecimento de indivíduos e comunidades aprisionados em disputas para o controle dos modos de viver e produzir.

## Introdução

O texto de Minayo ^1^ apresentando os conceitos e tipologias de violência abre a coletânea Impactos da Violência na Saúde ^2^ e nos ajuda a situar a caminhada que levou a Organização Mundial da Saúde (OMS), em 1996, e, em 2001, o Sistema Único de Saúde (SUS) a incluírem a violência na agenda da Saúde ^3^. Para a OMS, a violência compreende “*o uso intencional da força física ou do poder, real ou em ameaça, contra si próprio, contra outra pessoa, ou contra um grupo ou uma comunidade, que resulte ou tenha grande possibilidade de resultar em lesão, morte, dano psicológico, deficiência de desenvolvimento ou privação*” ^4^ (p. 5).

Em 1971, Dom Pedro Casaldáliga denunciava, pela primeira vez em carta pastoral ^5^, as diversas formas de violência contra comunidades na defesa pelo direito à preservação do modo de viver e produzir, direito esse presente na Amazônia por séculos ^6^ ^,^ ^7^ ^,^ ^8^: o acesso à terra. Esse quadro de violência persiste na região, que, em 2022, registrou aproximadamente 60% dos conflitos no campo ocorridos no Brasil ^9^. Em 2017, quatro dos cinco massacres no campo ocorreram na Amazônia e resultaram na morte de 25 camponeses ^10^. Os registros da Comissão Pastoral da Terra (CPT) apontam o recrudescimento dessas situações de conflitos nos últimos anos e a necessidade de se pensar a dimensão do sofrimento das pessoas reais por trás dos números ^9^. Para além dos danos e óbitos, impactos mais visíveis, os conflitos também se manifestam por meio de ameaças contra pessoas ou ocupações, resultando em impactos indiretos, os quais afetam o bem-estar físico e mental de indivíduos e populações e ampliando o desafio para a saúde pública na região ^11^ ^,^ ^12^.

A literatura sobre conflitos agrários na Amazônia é ampla e, em geral, descreve os conflitos como uma questão complexa e multifacetada. Conflitos são retratados como consequência da combinação de fatores relacionados à estrutura fundiária, à narrativa sobre “modernização” do campo, às estratégias desenvolvimentistas das três esferas de governo e ao conjunto de atividades ilícitas associadas às atividades econômicas que, em suas cadeias, envolvem grilagem, exploração ilegal mineral e madeireira ^13^ ^,^ ^14^ ^,^ ^15^ ^,^ ^16^.

Ancorado na sociologia rural, na história econômica da Amazônia e a partir de empirias e estudos de casos nela própria, o economista Francisco de Assis Costa centraliza esse debate na perspectiva da economia agrária regional ao estabelecer os conflitos fundiários como a dimensão mais visível do antagonismo dos dois principais projetos em disputa para o desenvolvimento de base rural em andamento na Amazônia ^6^ ^,^ ^17^. Tais projetos baseiam-se essencialmente na evolução de dois paradigmas associados aos sistemas técnicos de produção presentes na economia agrária amazônica: um cujo fundamento tecno-produtivo principal é a conservação e manutenção da integridade e diversidade do bioma, e o outro que é apoiado no padrão homogeneizador, que tem na exploração dos recursos naturais do bioma sua principal estratégia de evolução.

Esses dois padrões mobilizam essencialmente as questões relativas ao uso da terra, à organização da força de trabalho e ao pacote de solução tecnológica e uso da biodiversidade internalizada em seus sistemas de produção. Ao serem combinados com diferentes agentes com racionalidades econômicas específicas (camponeses e patronais), emergem diversos sistemas de produção rural que podem ser organizados para estabelecer, no contexto da categoria econômica analítica desenvolvida por Costa ^6^ ^,^ ^18^, as trajetórias tecnológicas ou trajetórias tecnoprodutivas rurais (TTs), um conjunto de seis trajetórias presentes na economia agrária amazônica.

Embora com nuances internas aos padrões de racionalidade camponesa e patronal, a base do pacote tecnológico homogeneizador que impulsiona (com base em soluções mecânico-químico-genéticas) a dinâmica produtiva rural na Amazônia ^19^, representa o principal motor das mudanças da cobertura florestal e o principal estímulo aos conflitos agrários. Por essa perspectiva, o recurso florestal e a população que reside na região são vistos como um empecilho, uma barreira para a ampliação da produção agropecuária da região. A ideia vem de um modo de produzir que utiliza os recursos naturais como uma natureza morta ^20^ ^,^ ^21^.

No paradigma alternativo, por sua vez, existe maior variedade de soluções tecnológicas, permitindo maior eficiência no uso do recurso florestal e dos valores culturais já estabelecidos pelas comunidades tradicionais na região, o qual é incorporado aos modos de produzir dos novos agentes econômicos que se transferem para o bioma, configurando um modelo de evolução tecnoprodutivo em moldes aproximados aos sistemas agroflorestais. O bioma é visto de maneira integrada, como um insumo produtivo, em que o modo de produzir depende da natureza viva, ou seja, do bioma em sua maior integridade. A diversidade estrutural e cultural é parte fundamental da organização da produção e da economia desses sistemas considerados social e ecologicamente mais saudáveis e resilientes ^22^ ^,^ ^23^, cujas zonas de produção não podem ser consideradas pré-capitalistas ^6^.

Na economia agrária, esses dois projetos ou paradigmas representam diferentes modelos de produção, reprodução e distribuição dos benefícios disponíveis, e as TTs associadas a eles se encontram em concorrência, ora em competição e ora em colaboração. Para o modelo de produção homogeneizador, a transformação da terra em mercadoria representa uma etapa essencial em todo o processo produtivo, pois é vista como um insumo produtivo. Assim, impõe-se um mercado de terras que as trata como uma mercadoria de caráter genérico, cuja produção ocorre pela conversão da floresta originária, um bem público, em patrimônio privado ^8^ ^,^ ^24^. É a floresta, primeiro, transformada em terra-com-mata, que é o insumo primário para a produção das terras-sem-mata: as “terras para a agricultura” ou as “terras para a pecuária” − constituindo-se em terras para o mercado ^17^. Em contrapartida, nas dinâmicas associadas aos sistemas camponeses, a terra-com-mata é um meio de produção que garante a economia da unidade de produção camponesa, de modo que há uma economia não-capitalista girando dentro de uma economia capitalista ^25^ ^,^ ^26^.

Assim, a demanda por terra-sem-mata (mercadoria terra) torna-se elemento constitutivo e mobilizador da dinâmica da economia agrária amazônica, estabelecendo um ponto de origem para os conflitos agrários. Uma economia que fomenta a concentração de terras e submete a população camponesa a diversas formas de violência e insegurança fundiária ^27^. Reiterando, Costa et al. ^28^ analisaram dados dos anos de 2016 a 2020 da Polícia Federal da Amazônia sobre a prevenção de crimes ambientais; foi observado que 47% dos imóveis investigados resultaram de grilagem e 60% apresentavam desmatamento ou exploração ilegal de madeira, demostrando que esse mercado de terras está interligado ao conjunto de atividades ilegais e, portanto, à violência e violação dos direitos humanos. Neste trabalho, assumimos que parte da terra acomodada no sistema agrário é, em algum nível, proveniente do ilícito, ou seja, proveniente de formas espoliativas de apropriação de terras, com uso de violência, resultando em transformações da paisagem florestal.

Uma forma de reconhecer no território onde atuam ou estão em curso os principais mecanismos geradores de terra, é a partir das situações de conflito mapeadas pela CPT, que desde 1985 registra o acumulado anual das situações de violências envolvendo as diversas categorias de camponeses no Brasil ^29^. Com base nesse acervo, é possível obter os conflitos ancorados no território, decorrentes diretamente da cadeia de produção dessas terras.

Neste trabalho, buscamos responder, utilizando uma abordagem de construção de evidências empíricas, por meio da integração de bases de dados espaciais, como as situações de conflito por terra são sinalizadoras da ação dos mecanismos geradores de terra que operam na Amazônia Legal. Nosso recorte para este estudo são os registros de conflitos por terra ocorridos na última década (2012 a 2021) na região. A análise abrange dois períodos. No primeiro, de 2012 a 2017, identificam-se e caracterizam-se os territórios onde os mecanismos geradores de terra estiveram atuantes. O estado associado à dinâmica do sistema de TTs em 2017, por meio de uma matriz de transições de 2006 para 2017, é observado em conjunto com os dados de conflitos. No segundo período, de 2018 a 2021, mostramos processos e tendências dos conflitos por terra para o período mais recente, considerando apenas o estado do sistema de TTs em 2017.

Assim, analisamos duas dinâmicas territorializadas no espaço regional: a dinâmica da ocorrência dos conflitos por terra e a dinâmica do sistema de TTs, conformando um instrumental analítico de base empírica. Os resultados contribuem para o debate sobre as violências na Amazônia a partir das escolhas pelo modelo de desenvolvimento agrário. Juntamente com o desfecho ambiental, observado pelo desmatamento, os conflitos por terra são um desfecho de saúde, revelando adoecimentos de indivíduos e comunidades, aprisionados em uma série de disputas assimétricas de poder pelo controle dos modos de viver e produzir de um bioma que historicamente evolui com a presença de comunidades e populações ancestrais.

## Materiais e métodos

### Área de estudo

A Amazônia Legal representa 58,93% do território brasileiro e inclui 772 municípios nas regiões Norte, Nordeste e Centro-oeste. Com 5 milhões de km^2^, a Amazônia Legal abriga 26.644.564 habitantes (13,12% da população brasileira) ^30^ e grande diversidade socioambiental ^7^ ^,^ ^8^. De 2012 a 2021, os nove estados da Amazônia Legal concentraram 63% dos 10.318 conflitos por terra ocorridos no Brasil, os quais envolveram aproximadamente 700 mil famílias e resultaram em 258 óbitos ^29^. Nesse período, os conflitos na região recrudesceram, culminando com 1.042 conflitos por terra em 2020, maior número registrado na série histórica anual da CPT. Paralelamente, a região observou o aumento da taxa de desmatamento, chegando a quase 13 mil km^2^ em 2021 e 2022 ^31^, e a expansão do agronegócio, com crescimento de 286% da área plantada de soja entre 2006 e 2017 ^32^.

### Materiais

A principal base de dados deste trabalho são as situações de conflito documentadas pela CPT e disponíveis no acervo do Centro de Documentação Dom Tomás Balduíno (CEDOC) ^29^. Para a CPT, conflitos são ações de resistência e enfrentamento que acontecem em diferentes contextos sociais no âmbito rural envolvendo a luta pela terra, água, direitos e pelos meios de trabalho e produção, e ocorrem entre classes sociais, entre os trabalhadores, ou pela ausência ou má gestão de políticas públicas ^29^. Por essa base ser continuamente atualizada, o número total de conflitos pode diferir do divulgado nos relatórios anuais.

Na base da CPT, os registros são categorizados em conflitos por terra, pela água e trabalhistas. Apenas os conflitos por terra foram analisados, ou seja, as ações de resistência e enfrentamento pela posse, uso e propriedade da terra e pelo acesso aos recursos naturais, envolvendo as mais diversas categorias de camponeses ^9^. Os conflitos são indexados pelo nome e cada ação de enfrentamento ou resistência é considerada uma ocorrência. Assim, em uma área, pode ocorrer mais de uma situação de conflito ao longo do ano. Cada conflito também é descrito quanto ao número de famílias afetadas, tipo de violência sofrida e agentes sociais envolvidos. O município de origem da ocorrência de cada conflito foi considerado para a indexação espacial e foram selecionados os conflitos ocorridos entre 2012 e 2021.

A segunda base de dados utilizada refere-se à base territorializada da economia agrária amazônida, mediada pelas TTs. Para identificar as TTs, Costa ^18^ desenvolveu um método baseado em regressões multivariadas e técnicas de análise de componentes principais e fatorial aplicadas aos dados extraídos dos últimos três Censos Agropecuários (1995, 2006 e 2017). Dessa abordagem, emergiram, para os municípios da Amazônia Legal, seis tipos de TTs categorizadas em camponesas (TT1, TT2 e TT3) e patronais (TT4, TT5&6 e TT7) e caracterizadas quanto aos modos de produzir, tipos de atividades, força de trabalho e pacote de soluções tecnológicas (Quadro 1). As participações percentuais das TTs em relação ao componente agrário do valor da produção municipal encontram-se disponíveis em Costa ^33^. Neste estudo, adotamos a TT dominante em cada município, ou seja, a TT que contribuiu com mais de 50% no valor total da contribuição derivada da economia rural para o Valor Bruto da Produção em 2006 e 2017.

**Quadro 1:** Descrição do conjunto de trajetórias tecnológicas (TTs) presentes na economia agrária amazônica.

	DESCRIÇÃO	TT	SISTEMAS PRODUTIVOS
Camponesas	Sistemas de produção baseados em estabelecimentos rurais geridos por arranjos de “base familiar”	TT1	Sistemas que convergem para culturas permanentes (cacau, pimenta, café) e temporárias (mandioca, milho, arroz e feijão) de pequena escala
		TT2	Sistemas agroflorestais
		TT3	Pecuária de corte e leiteira de pequena escala e culturas temporárias e permanentes
Patronais	Estabelecimentos geridos pelo regime do “agronegócio” ou “assalariado”, nos quais as decisões microeconômicas estão alinhadas com os grandes mercados e o comércio de *commodities*	TT4	Pecuária de grande escala e produção de culturas para consumo animal
		TT5&6	Grandes lavouras de culturas permanentes, florestas plantadas e silvicultura tecnificada
		TT7	Sistemas intensivos de culturas temporárias em larga escala (soja, arroz, milho, etc.)

### Procedimentos metodológicos

Neste estudo exploratório de base territorializada, a unidade espacial de análise são os municípios da Amazônia Legal, os quais foram associadas às situações de conflitos por terra entre 2012 e 2021 e às categorias analíticas da economia agrária (TTs) para os anos de 2006 e 2017. Adotamos o ano de 2017 como referência dada a classificação mais recente das TTs, dividindo a década em dois períodos de análise. De 2012 a 2017, buscamos identificar e caracterizar os territórios de atuação dos mecanismos geradores de terra no período que culminou com as TTs encontradas em 2017. De 2018 a 2021, pretendemos localizar os processos e as tendências associadas aos conflitos por terra, mantendo como referência a categorização das TTs em 2017, considerando que esses conflitos se originam nessas categorias ou são produzidos pelas transições que as envolvem.

A dinâmica evolutiva do sistema de TTs observou o estado do sistema em 2006 e em 2017, visando detectar as transições de mudança e de não mudança para cada município, ou seja, se a TT dominante em 2006 permanecia dominante em 2017 ou se houve uma mudança de estado, indicando a natureza da transição. Uma matriz das transições de estado das TTs entre 2006 e 2017 foi construída e os quantitativos das ocorrências de conflitos agregados para o período de 2012 a 2017 foram associados às transições identificadas. Para o período de 2018 a 2021, os acumulados dos conflitos foram integrados com o estado do sistema de TTs observado no ano de 2017.

Complementarmente, exploramos a hipótese de dependência espacial associada às ocorrências de conflitos por terra para os períodos analisados. Foram utilizados índices que medem a autocorrelação espacial, global e local, são eles: Índices de Moran Global e Local e um quadro exploratório baseado em Indicadores Locais de Associação Espacial Local (LISA, acrônimo em inglês) ^34^. Enquanto indicadores globais fornecem uma única medida de associação espacial para todo o conjunto de dados, indicadores locais produzem um valor específico para cada município, permitindo a identificação e caracterização de aglomerados municipais. O LISA é uma decomposição do índice de Moran Global, calculado para cada município, que permite representar na forma de mapa a estrutura de dependência espacial em nível local.

Dessa análise resultam quatro tipos de aglomerados espaciais municipais: Alto-Alto agrupa municípios com alta ocorrência de conflitos e cuja vizinhança também possui municípios com alta ocorrência desses eventos no mesmo período; Baixo-Baixo agrupa municípios com baixa ocorrência de conflitos cuja vizinhança também apresenta baixa ocorrência no mesmo período; e Alto-Baixo e Baixo-Alto são indicativos de municípios com alta/baixa ocorrência de conflitos, cercados por municípios com ocorrência em situação contrária. Para obter os índices, foi utilizado o software GeoDa (https://spatial.uchicago.edu/geoda) ^35^ e a pseudo-significância executada com 999 permutações aleatórias.

## Resultados

### A geografia dos conflitos por terra na Amazônia na década 2012-2021

De 2012 a 2021, o CEDOC/CPT contabilizou 6.505 conflitos por terra nos nove estados da Amazônia Legal. Anualmente, os conflitos por terra apresentaram relativa estabilidade entre 2012 e 2015, com média anual de 475,5 conflitos; pico em 2016, quando foram registrados 764 conflitos e média anual de 682,3 conflitos no período compreendido entre 2016 e 2018; e número elevado de conflitos entre 2018 e 2021, com média anual de 852. Em 2020, o número de conflitos por terra (1.042) foi impactado pela pandemia da COVID-19, devido principalmente à omissão e à conivência do Estado perante as situações de exposição e vulnerabilidade de muitas famílias ^36^. De 2019 a 2021, foram contabilizados 2.552 conflitos (39,2%).

Maranhão, Pará e Rondônia concentraram 57,1% (3.714 conflitos) do total de conflitos. Em termos relativos, ao longo do período analisado, a contribuição do Maranhão (de 33,3%, em 2012, para 13,9%, em 2021) e do Amapá (11,7%, em 2012, para 7%, em 2021), no total de conflitos registrados na Amazônia Legal, diminuiu, enquanto a participação do Mato Grosso (de 5,5%, em 2012, para 12,7%, em 2021), do Pará (de 17,5%, em 2012, para 22,6%) e de Roraima (nenhum conflito em 2012 para 7,4% em 2021) aumentou. Em Roraima, o pico de conflitos em 2021, que representou 10,9% (113) do total ocorrido na Amazônia Legal, refere-se às violências ocorridas contra os territórios e povos indígenas.

Em relação aos responsáveis pelos conflitos, conforme categorização da CPT ^29^, fazendeiros responderam por 31,9% (2.073), seguidos por grileiros com 17,5% (1.140) e empresários com 16,1% (1.047) dos conflitos. Fazendeiros é a categoria que mais causou conflitos na maioria dos estados: Acre (64,1%, 378), Rondônia (51%, 419), Mato Grosso (44,4%, 283), Tocantins (31,3%, 136), Maranhão (28,3%, 469) e Amazonas (26,3%, 103). Também teve participação expressiva no Pará, sendo responsável por 20,2% (249) das ocorrências. Os grileiros provocaram cerca de 30% dos conflitos ocorridos no Tocantins (132) e no Pará (366), 21,7% (85) no Amazonas e cerca de 15% no Mato Grosso (107), Rondônia (101) e Maranhão (249). Os conflitos causados por empresários foram especialmente relevantes no Amapá, representando 67,9% (381) dos conflitos e no Maranhão (23,6%, 392). Os conflitos causados pelo Estado e agentes da lei, em suas diferentes esferas, ocorreram em todos os estados, sendo mais significativos em Roraima (26,1%) e no Tocantins (25,1%). Para Roraima, os dados refletem a relação conflituosa entre o garimpo e os povos indígenas e seus territórios: 58% dos conflitos ocorridos no estado foram provocados por garimpeiros e em 72,7% os indígenas foram as vítimas. Em toda a Amazônia Legal, os indígenas foram alvos em 1.216 conflitos (18,7%), especialmente no Pará (299), Mato Grosso (290) e Maranhão (146).

Os posseiros representam a categoria que mais sofreu violências no período analisado: 1.679 conflitos (25,8%), sendo: 60,6% (340) do total de conflitos ocorridos no Amapá, 40% (236) no Acre e 37,6% (623) no Maranhão. Já os conflitos envolvendo os sem-terra totalizaram 1.249 ocorrências (19,2%), das quais 40,3% (504) foram no Pará e 29,2% (365) em Rondônia. No Maranhão, 37,6% dos conflitos registrados foram contra quilombolas, enquanto no Acre, seringueiros foram vítimas em 48% das ocorrências.

### A economia agrária e os conflitos por terra na Amazônia

Entre 2012 e 2017, foram registradas 3.326 (51,12% do total na década) ocorrências de conflitos por terra na Amazônia Legal distribuídas em 386 municípios. Destes, 1.700 conflitos ocorreram em municípios com mudanças no estado da TT dominante de 2006 para 2017, os quais representam 56,04% (436) do total de municípios compreendidos na base de dados produzida por Costa ^33^ (Figura 1a).

**Figura 1:** Espacialização dos conflitos (2012 a 2017) e das transições das trajetórias tecnológicas (TTs) dominante (2006 para 2017) e matriz com a distribuição do acumulado de conflitos (2012 a 2017) pelos tipos de transições de estado da TT dominante (2006 a 2017).

* Transições com maior número de ocorrências de conflitos por terra (2012 a 2017).

A TT7, associada à agricultura patronal, expandiu seus domínios em mais de 70%, passando de 102 (13%), em 2006, para 176 (23%) municípios, em 2017, enquanto a expansão da TT4, pecuária patronal, foi de quase 20%, passando de 216 (28%) para 259 (33%) municípios. Tais TTs se mantiveram em mais de 65% dos municípios. As transições associadas a essas duas, seja em situação de permanência (não mudança) ou de mudança, concentraram 61,25% (1.889) dos conflitos por terra registrados entre 2012 e 2017 (Figura 1b). Desse montante, 558 (29,5%) estão associados a municípios de não mudança da TT4; 399 (21,12%) em transições de TT3, camponesa com especificidade em produção pecuária, para TT4, patronal pecuária que atua em concorrência direta com a TT3; e 229 (12,12%) em municípios de não mudança da TT7. Essas transições ocorrem principalmente ao longo de uma faixa contínua, abrangendo o sul maranhense, sul-sudeste paraense, Tocantins, Mato Grosso, Rondônia até o leste acreano (Figura 1a).

Em contrapartida, as transições relacionadas às TTs camponesas responderam por 34,5% (1.064) dos conflitos do período e três transições se destacam: não mudança da TT3, com 310 conflitos registrados (29,1%; 41 municípios); não mudança da TT2, camponesa de sistemas agroflorestais, com 174 conflitos (16,4%; 43 municípios); e mudança da TT2 para a TT1, camponesa com agricultura especializada, com 167 conflitos (15,6%; 68 municípios).

Algumas dessas transições emergiram nos agrupamentos espaciais de municípios Alto-Alto para a ocorrência de conflitos, no período de 2012 a 2017, como na região da AMACRO (acrônimo para a região de fronteira do Amazonas, Acre e Rondônia) e no sudeste paraense (Figura 2). A AMACRO, nomeada mais recentemente como Zona de Desenvolvimento Sustentável (ZDS) Abunã-Madeira ^37^, é uma das principais fronteiras ativas e recentes de desmatamento na Amazônia Legal ^38^, com processos de desterritorialização de povos tradicionais ^39^. O sudeste paraense, em especial São Félix do Xingu, representa a persistência das violências em fronteiras históricas ^14^. Conflitos associados à constituição da propriedade privada por meio da grilagem e expansão do agronegócio e à implantação de empreendimentos, como hidrelétricas ao longo do rio Araguari (Amapá), conformaram o agrupamento de municípios Alto-Alto no Amapá e norte do Pará. Estão nesse agrupamento municípios que convergiram para a TT7 em 2017, como Tartarugalzinho, Ferreira Gomes e Itaubal (Amapá), bem como municípios que permaneceram em TTs camponesas, como Mazagão, Macapá (Amapá) e Afuá (Pará).

**Figura 2:** Agrupamentos espaciais de municípios quanto à ocorrência de conflitos para 2012 a 2017 * e 2018 a 2021 **.

* (I: 0,206; p < 0,001); ** (I: 0,148; p < 0,001); *** Significativos a 95%.

No período seguinte (2018 a 2021), emergiram cinco agrupamentos espaciais de municípios Alto-Alto (Figura 2). Novamente, destaca-se a AMACRO e São Félix do Xingu, cujo agrupamento expande incorporando municípios como Altamira, Novo Progresso, Itaituba e Jacareacanga (Pará). O agrupamento no Amapá persiste, com menos municípios que no período anterior. No noroeste paraense, Óbidos, Monte Alegre e Alenquer emergem como Alto-Alto, devido às violências contra as populações quilombolas e seus territórios. O *cluster* Alto-Alto em Roraima é explicado pelos conflitos nos territórios indígenas, especialmente a partir de 2020. Nos dois períodos, destaca-se a persistência de *cluster* Baixo-Baixo no Amazonas, nos municípios de Jutaí e Codajás.

Para esse período, de 2018 a 2021, foram registrados 3.179 conflitos (48,87% do total), distribuídos em 396 municípios (Figura 3). Os municípios cujo estado da TT dominante em 2017 era camponesa representaram 35,8% das ocorrências de conflitos. Municípios com TT1 dominante registraram 379 conflitos (11,9%); na TT2, 315 conflitos (9,9%), enquanto na TT3 foram registrados 445 (14%) conflitos. Já os municípios associados à TT4 concentraram quase 40% (1.231) do total de conflitos registrados na Amazônia. Nos municípios em que as TT5&6 é dominante, foram associados 106 conflitos (3,3%) e aos da TT7, 574 conflitos (18,1%). 

**Figura 3:** Distribuição dos conflitos por terra por município acumulados para os períodos de 2012 a 2017 e 2018 a 2021 e o estado das trajetórias tecnológicas (TTs) em 2017.

A análise integrada dos acumulados dos conflitos nos municípios nos dois períodos revela três dinâmicas em curso. A primeira refere-se à dispersão dos conflitos, que entre 2012 e 2017 estavam concentrados em municípios principalmente do Amapá, Maranhão, sudeste do Pará, Rondônia e Acre. O número de municípios com registros de conflitos por terra passou de 386 para 396 municípios no segundo período. Seguindo a dispersão dos conflitos, ocorre a interiorização das violências em direção à Amazônia ocidental. São observados municípios com alto número de ocorrências no oeste paraense e em Roraima, bem como a ocorrência de conflitos em novas áreas de municípios na porção oeste da Amazônia Legal, especialmente no interior do estado do Amazonas. A terceira dinâmica está associada à intensificação da ocorrência de conflitos em municípios no Mato Grosso, nos quais predominam sistemas patronais de produção de grãos.

## Discussão

Na Amazônia, a composição de atores sociais envolvidos nas situações de violência em cada estado varia a depender do contexto e processo histórico-geográfico de ocupação, cujos desfechos em saúde transcendem aspectos meramente clínicos, ameaçando populações e seus modos de viver e (re)produzir. A diversidade de agentes ilícitos promotores dessas violências inclui milícias, traficantes e pistoleiros ^29^. Para Costa et al. ^28^, o mercado de terras na Amazônia está intrinsecamente ligado ao que foi chamado de “coalizão da pilhagem”, ou seja, uma aliança histórica entre as elites fundiárias, atores econômicos poderosos, políticos influentes e organizações criminosas para explorar os recursos na região, por meio de atividades legais e ilegais, com variadas formas de violência. Schmink et al. ^14^ oferecem, a partir do Município de São Félix do Xingu, um panorama dos conflitos e das transformações na fronteira, desde 1970, produzidos pela interação entre um conjunto de atores sociais em evolução com os debates e mudanças de políticas em diferentes níveis.

Bruno ^40^ reflete sobre a roupagem da “modernização” e incorporação de novos símbolos nas narrativas das elites agrárias, enquanto se mantêm inscritas em um mesmo espaço de referências e de significações, que incluem violências, crime ambiental, lucro e concentração de terras. No período analisado, municípios dominados pelos sistemas patronais foram responsáveis por quase 4 mil conflitos por terra (61,5% do total), concentrados principalmente nas regiões dominadas pela pecuária de larga escala (TT4), em que ocorreu quase 50% (37.417,05km^2^) do desmatamento na Amazônia Legal no mesmo período. As TTs 4 e 7 apresentam maior peso na dinâmica fundiária na Amazônia Legal, juntas representaram 78% (12,3 milhões de hectares) dos acréscimos de terras (mercadoria) anotadas nos censos agropecuários de 2006 e 2017 ^17^.

Municípios dominados pela TT4 também estão associados a áreas de persistência de conflitos identificados pelos agrupamentos espaciais. Essas áreas ocorrem em fronteiras históricas, como no sul-sudeste paraense ^14^, bem como nas novas frentes, como na AMACRO ^39^. Compreendendo 32 municípios, a AMACRO foi responsável por quase 30% do desmatamento detectado entre 2012 e 2022 ^31^. Essa região também é alvo da grilagem e de projetos para a expansão do agronegócio, articulada por empresários do setor em parceria com o Estado ^38^. Assim como na AMACRO, o sul-sudeste paraense se destaca pelo desmatamento, com municípios entre os 10 maiores desmatadores da Amazônia ^31^. O eixo associado à BR-163, que engloba parte desses municípios, é também uma área de expansão da fronteira agropecuária, com recente ampliação das áreas destinadas à agricultura de grãos ^41^.

Contudo, é na TT7 que a roupagem da modernização está especialmente circunscrita às narrativas. Os municípios dominados pela TT7 registraram cerca de 18% dos conflitos por terra e correspondem a 11% do desmatamento ^31^. Adicionalmente, no segundo período, há uma dinâmica de intensificação das ocorrências no Mato Grosso, com forte presença de sistemas produtivos associados à TT7. Esses resultados corroboram com uma literatura crescente, mas não dominante, que discute as tensões associadas ao agronegócio. Nesse caso, a baixa demanda de mão de obra, devido ao sistema de monocultivo, mecanizado com uso de insumos químicos e genéticos, contribui para o êxodo rural, o crescimento desordenado das periferias urbanas, a redução da capacidade de produção de alimentos tradicionais ^27^ ^,^ ^42^ e o adoecimento de populações decorrente do uso e espalhamento de agrotóxicos ^43^. No entorno de Santarém, por exemplo, a expansão da agricultura mecanizada ocorreu principalmente em terras de produção camponesa e ribeirinha, resultando na homogeneização da paisagem e na perda florestal nas áreas de produção e no seu entorno ^44^.

Nossos resultados mostram a dinâmica de interiorização dos conflitos por terra para municípios agroflorestais na Amazônia ocidental, observada entre 2018 e 2021, revelando novas frentes ^45^ de interesse da economia agrária pelo recurso terra. Sumariamente, esses municípios se caracterizam pela maior presença do recurso florestal, pelo significativo estoque de terras sem destinação fundiária ^46^ ^,^ ^47^ e pela manutenção de economias baseadas no bioma em arranjos diversos, comandadas principalmente pela TT2, com desmatamento baixo ou quase zero ^31^. Assim, diferentemente das áreas onde o desmatamento é um fato consumado, o conflito por terra, nessas novas frentes, pode ser visto como indicativo de um estágio anterior ao avanço da fronteira agropecuária, quando o mercado de terras é instaurado, mas sem provocar mudanças significativas na paisagem florestal. Visto dessa forma, o conflito por terra sinaliza no território que há uma disputa entre os modelos de desenvolvimento, ou seja, controlar essas disputas e o acesso ilícito à terra nesse estágio é primordial, sob o risco do agravamento da violência, do processo de perda florestal, que tem se mostrado irreversível em frentes de ocupação mais antigas ^45^, e de todos os prejuízos diretos e indiretos que esse processo desencadeia nas condições de saúde e bem-estar das populações locais. Na medida em que as lógicas patronais se expandem sobre os territórios campesinos e a floresta, desterritorializam-se esses sistemas e suas populações, que têm no bioma, em sua maior integridade, os meios para a reprodução social do modo de vida amazônico ^48^.

Retornando à provocação “a violência faz mal à saúde”, apresentada em Minayo ^1^, os impactos dos conflitos por terra na Amazônia têm sua dimensão mais visível nas lesões e nos óbitos. Contudo, para além da violência física, os conflitos sistematizados pelo CEDOC/CPT reúnem também as ameaças contra pessoas e a ocupação. Em conjunto, essas violências resultam em impactos variados, cuja existência e extensão não são totalmente conhecidas, os quais afetam a qualidade de vida e o bem-estar físico e mental de indivíduos e populações. A recente crise nos Yanomamis (Roraima), deflagrada pelo garimpo, mesmo com o reconhecimento de sua identidade e territórios, homologados há mais de 30 anos, ilustra a complexidade dos impactos resultantes dos conflitos que afetam as condições de saúde da população ^49^ ^,^ ^50^, cujas medidas para mitigar e evitar sua ocorrência representam também a promoção e a proteção à vida, inclusive para gerações futuras.

Como limitações deste estudo, citamos aquelas associadas às unidades espaço-temporais de análise e à abordagem metodológica. A agregação dos conflitos por terra à escala municipal pode mascarar dinâmicas e processos locais explicativos da ocorrência de conflitos, bem como os impactos nas condições de saúde das populações locais. Em relação ao método, a análise exploratória em nível municipal pode ter sua robustez aperfeiçoada pela inclusão de dados espaciais, especialmente relacionados à estrutura de terras e fundiária, combinados em abordagens quantitativas. Além disso, por fim, uma melhor caracterização dos conflitos nas categorias que estabelecem o campesinato, com o tratamento da base de dados da CPT, para aperfeiçoar as análises das dinâmicas observadas e a sinalização para os desfechos relativos à saúde, à perda de cobertura florestal e à sociobiodiversidade do bioma.
